# Improving the active expression of transglutaminase in *Streptomyces lividans* by promoter engineering and codon optimization

**DOI:** 10.1186/s12896-016-0304-7

**Published:** 2016-10-28

**Authors:** Song Liu, Miao Wang, Guocheng Du, Jian Chen

**Affiliations:** 1 Key Laboratory of Industrial Biotechnology, Ministry of Education, School of Biotechnology, Jiangnan University, Wuxi, China; 2 National Engineering Laboratory for Cereal Fermentation Technology, Jiangnan University, Wuxi, China; 3 Key Laboratory of Carbohydrate Chemistry and Biotechnology, Ministry of Education, School of Biotechnology, Jiangnan University, Wuxi, China; 4 School of Food Science and Technology, Jiangnan University, Wuxi, 214122 China

**Keywords:** Transglutaminase, Endogenous promoter, Codon optimization, *Streptomyces hygroscopicus*, *Streptomyces lividans*

## Abstract

**Background:**

Transglutaminases (TGase), which are synthesized as a zymogen (pro-TGase) in *Streptomyces* sp., are important enzymes in the food industry. Because this pro-peptide is essential for the correct folding of *Streptomyces* TGase, TGase is usually expressed in an inactive pro-TGase form, which is then converted to active TGase by the addition of activating proteases in vitro. In this study, *Streptomyces hygroscopicus* TGase was actively produced by *Streptomyces lividans* through promoter engineering and codon optimization.

**Results:**

A gene fragment (*tg1*, 2.6 kb) that encoded the pro-TGase and its endogenous promoter region, signal peptide and terminator was amplified from *S. hygroscopicus* WSH03-13 and cloned into plasmid pIJ86, which resulted in pIJ86/*tg1*. After fermentation for 2 days, *S. lividans* TK24 that harbored pIJ86/*tg1* produced 1.8 U/mL of TGase, and a clear TGase band (38 kDa) was detected in the culture supernatant. These results indicated that the pro-TGase was successfully expressed and correctly processed into active TGase in *S. lividans* TK24 by using the TGase promoter. Based on deletion analysis, the complete sequence of the TGase promoter is restricted to the region from −693 to −48. We also identified a negative element (−198 to −148) in the TGase promoter, and the deletion of this element increased the TGase production by 81.3 %, in contrast to the method by which *S. lividans* expresses pIJ86/*tg1*. Combining the deletion of the negative element of the promoter and optimization of the gene codons, the yield and productivity of TGase reached 5.73 U/mL and 0.14 U/mL/h in the recombinant *S. lividans*, respectively.

**Conclusions:**

We constructed an active TGase-producing strain that had a high yield and productivity, and the optimized TGase promoter could be a good candidate promoter for the expression of other proteins in *Streptomyces*.

## Background

Transglutaminase (TGase, EC 2.3.2.13) is an enzyme that exhibits several catalytic activities: the crosslinking of proteins by forming N^ε^-(γ-glutamyl) lysine bonds, the incorporation of polyamines into protein, and the deamidation of protein-bound glutamines [1]. Because of these catalytic abilities, TGase has been widely used in industrial processing, especially in food processing, for improving the functional properties of various proteins, including meat, soy, myosin, globulin, casein, peanut, and whey proteins [2]. TGase is widely distributed in various organisms, including plants [3], mammals [4], and microorganisms [5]. Among the TGases, the TGase from *Streptomyces* is Ca^2+^-independent and is advantageous for industrial applications because it has a higher reaction rate, broad substrate specificity for an acyl donor, and a smaller molecular size [6, 7]. The development of an efficient and easy-to-use expression system for the production of *Streptomyces* TGase is therefore highly desirable.

*Streptomyces* TGase is secreted as pro-TGase and becomes active after the cleavage of the pro-peptide by endogenous activating proteases [5]. Because the pro-peptide is essential for the correct folding of TGase, direct expression of mature TGase yields insoluble inclusion bodies [8] or inactive enzyme [9]. Thus, TGase is usually expressed in a pro-TGase form [10, 11]. Due to the absence of activating protease in the host strain, co-expression of heterologous proteases is required to convert pro-TGase into active TGase [12]. Because of the ability to convert the pro-protein into the active enzyme with its own proteases, *Streptomyces* hosts became ideal hosts for producing active TGase. TGases from *Streptoverticillium mobaraense*, *Streptoverticillium ladakanum*, and *Streptomyces platensis* have been heterologously expressed in *Streptomyces lividans* as an active enzyme [13–15]. However, the secretion level of TGase in *S. lividans* 3131 is less than 0.01 U/mL [13]. When *S. lividans* JT46 was used as the host strain, the yield of TGase reaches only 1.23–2.22 U/mL after 3–6 days of fermentation [14, 15]. Overall, both the yield and productivity of TGase as expressed in *Streptomyces* hosts are still low.

The *ermE* and *tipA* promoters have proven to be highly successful for the over-expression of *Streptomyces* genes [16]. However, the *ermE* promoter improved TGase production by 0.8 U/mL [17], and there are no reports for TGase expression with the other strong promoters. It has been found that the endogenous promoter of TGase is recognized in *S. lividans*, and the yield of the recombinant *S. platensis* TGase reached 2.22 U/mL [15], which suggests that the endogenous promoter of different TGases or its modified versions could be more efficient for TGase expression by *S. lividans* in contrast to heterologous strong promoters*.* In addition, the *Streptomyces* genome has a high (>70 %) GC content, and rare codons such as TTA could significantly reduce the protein expression in *S. lividans* [18]. However, the *Streptomyces* TGase gene contains rare codons such as TTA, although it was found in *Streptomyces* [14, 15, 19]. Thus, codon optimization could also benefit TGase expression in *S. lividans.*

Previously, we cloned the DNA fragment (GenBank No: HM231108) that contained the TGase gene with a flanking region sequence from the *S. hygroscopicus* genome, and a putative promoter region was found upstream to TGase [10]*.* In this study, the *S. hygroscopicus* TGase gene was expressed in *S. lividans* TK24 by using its putative endogenous promoter. Then, the putative promoter was partially deleted, and the effects of the deletions on the expression of TGase in *S. lividans* TK24 were analyzed. In addition, the codons of TGase were optimized to further enhance the level of TGase expression. Finally, a relatively high level of TGase expression in *S. lividans* was achieved.

## Results

### Expression of TGase in *S. lividans* using its endogenous promoter

**Fig. 1:**
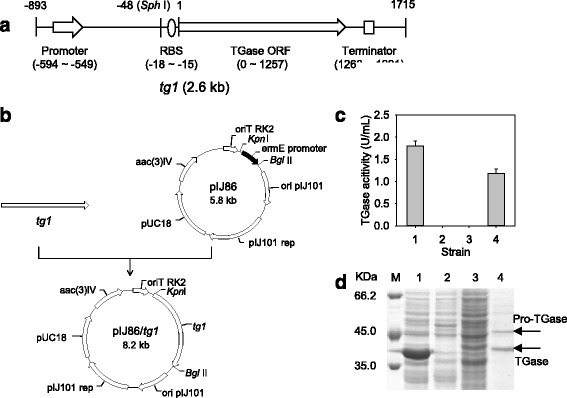
Production of TGase by *S. lividans* TK24/pIJ86/*tgl*. **a** The gene structure of *tg1.* **b** Construction of TGase expression plasmid pIJ86/*tg1*. **c** TGase activity assay of the culture supernatants of *Streptomyces* strains. **d** SDS-PAGE analysis of the TGases in the culture supernatants of *Streptomyces* strains. Labeling for (**a**): The numbers in the illustration indicate the base positions. Labeling for (**c**): 1: *S. lividans* TK24/pIJ86/*tgl* (the recombinant strain that expresses the *S. hygroscopicus* TGase ORF using the TGase endogenous promoter), 2: *S. lividans* TK24/pIJ86 (the control strain that carries pIJ86), 3: *S. lividans* TK24 (the control strain without the expression plasmid), 4: *S. hygroscopicus* WSH03-13 (the wild type strain that produces TGase). Labels for (**d**): M: protein marker, 1: *S. lividans* TK24/pIJ86/*tgl*, 2: *S. lividans* TK24/pIJ86, 3: *S. lividans* TK24, 4: *S. hygroscopicus* WSH03-13. The recombinant *S. lividans* TK24 were inoculated into 30 mL of medium (which contained 50 μg/mL apramycine) and cultured at 30 °C and 200 rpm for 48 h

To express the TGase in *S. lividans* using its endogenous promoter, a gene fragment (*tg1*, 2.6 kb) was amplified from the *S. hygroscopicus* genome (Fig. 1a) and cloned into pIJ86, which resulted in the plasmid pIJ86/*tg1* (Fig. 1b). The *tg1* encoded the TGase ORF (1257 bp), the upstream sequence (893 bp) and the downstream sequence (458 bp) (Fig. 1a). As analyzed previously, the *S. hygroscopicus* TGase ORF was composed of a secretory signal peptide gene, a pro-peptide gene, and the mature TGase gene; the upstream and downstream sequence of the ORF contain a putative promoter and a putative terminator, respectively [10]. The expression vector was transformed into *S. lividans* TK24, yielding *S. lividans* TK24/pIJ86/*tg1*.

When cultivated for 48 h, *S. lividans* TK24/pIJ86/*tg1* obtained 1.8 U/mL of extracellular TGase, which was approximately 1.5-fold of that achieved in the wild strain *S. hygroscopicus* WSH03-13 under the same cultivation conditions (Fig. 1c). TGase activity was not detected in the culture supernatants of the control strains *S. lividans* TK24/pIJ86 (*S. lividans* TK24 carrying pIJ86) and *S. lividans* TK24 (Fig. 1c). After treatment with TGase-activating protease dispase [10], the culture supernatants of the control strains still did not exhibit TGase activity (data not shown). Then, the culture supernatants of *S. lividans* TK24/pIJ86/*tg1*, *S. hygroscopicus*, and the control strains were subjected to SDS-PAGE analysis. As shown in Fig. 1d (lane 1), the *S. lividans* TK24/pIJ86/*tg1* showed a remarkable band that had a size of 38 kDa, which corresponds to the molecular weight of *S. hygroscopicus* TGase [20]. In the case of control strains, a small number of TGase/pro-TGase-like bands was detected in the culture supernatants (Fig. 1d, lanes 2 and 3). For failing to detect TGase activity in the control samples (Fig. 1c), these TGase/pro-TGase-like bands could correspond to the endogenous extracellular proteins of *S. lividans* TK24. Two proteins with approximate molecular weights of pro-TGase and TGase were detected in the culture supernatants of *S. hygroscopicus* (Fig. 1d, lane 4), which indicates that pro-TGase is not fully processed [10]. Because the *ermE* promoter was removed in pIJ86/*tg1*, our results indicated that the upstream sequence (893 bp) contains the endogenous promoter, which could drive the expression of TGase in *S. lividans* TK24. Moreover, the pro-TGase is correctly processed by the host proteases, which suggests that *S. lividans* TK24 is an ideal host for the active expression of TGase.

### Deletion analysis of the TGase promoter

As shown in Fig. 1a, the putative core promoter was located in the upstream sequence (between −594 bp and −549 bp) of the TGase ORF. To identify the TGase promoter, we analyzed the effect of the upstream sequence deletions on the expression of TGase in *S. lividans* TK24.

**Fig. 2:**
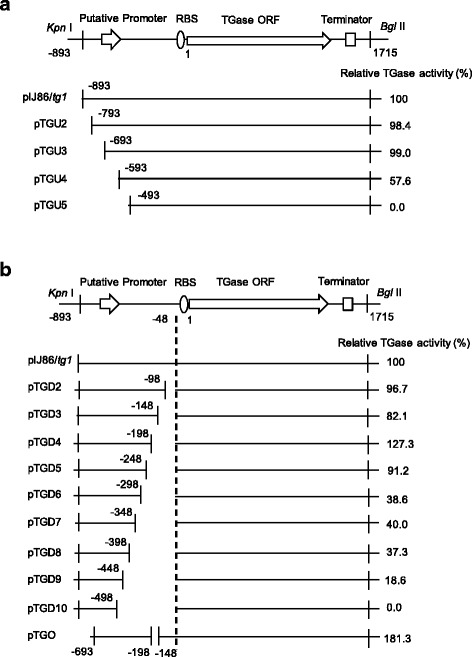
The effect of the endogenous promoter modification on the expression of TGase in *S. lividans*. **a** Partially deleting the 5′-end of the TGase promoter region. **b** Partially deleting the 3′-end of the TGase promoter region. The recombinant *S. lividans* TK24 were inoculated into 30 mL of medium (which contained 50 μg/mL apramycine) and cultured at 30 °C and 200 rpm for 48 h

First, deletions at the 5′-end of the upstream sequence were conducted. Deleting the upstream down to −793 (pTGU2) or −693 (pTGU3) had no significant effect on the expression level of the TGase gene (Fig. 2a). However, deleting the upstream down to −593 (pTGU4) resulted in a significant decrease in the TGase production, approximately 57.6 % of the activity of non-deletion (Fig. 2a). Deleting down to −493 (pTGU5) resulted in the complete loss of the TGase activity (Fig. 2a). These results suggest that the region from −693 to −493 contains important components of the TGase promoter.

Second, deletions at the 3′-end of the upstream sequence were conducted. Because the putative ribosome-binding site was located in the upstream sequence between −18 and −15, the 3′-end deletion was initiated at −48. Deleting upstream from −48 up to −98 (pTGD2) did not have a significant effect on the expression level of the TGase gene (Fig. 2b). Deletion of up to −148 (pTGD2) resulted in a decrease in the TGase activity, approximately 82.1 % of the activity of the non-deletion (pIJ86/*tg1*) (Fig. 2b). Interestingly, deleting up to −198 (pTGD4) increased the TGase activity by 27.3 % (Fig. 2b). However, the deletion mutant pTGD5 resulted in a decrease in the TGase activity (91.2 %), and further deletion (pTGD6-pTGD10) caused a significant decrease in the TGase activity (less than 40 % of the activity of pIJ86/*tg1*) (Fig. 2b). Last, TGase activity could not be detected with the deletion mutant pTGD10 (−498) (Fig. 2b). These results suggest that the region from −498 to −198 and the region from −148 to −98 could be the positive elements for the TGase promoter, while the region from −198 to −148 was the negative element for the TGase promoter.

Based on the deletion analysis (Fig. 2), the complete promoter of TGase could be restricted to the sequence from −693 to −48 in *tg1*. Because the region from −198 to −148 negatively affected the expression, this region was deleted from the complete promoter (−693 to −48), which yielded the TGase expression plasmid pTGO. As indicated by Fig. 2b, cells that carried pTGO achieved 3.3 U/mL of TGase, which was 81.3 % higher than that obtained by pIJ86/*tg1* (Fig. 2b).

### Codon optimization of the TGase gene in *S. lividans*

**Fig. 3:**
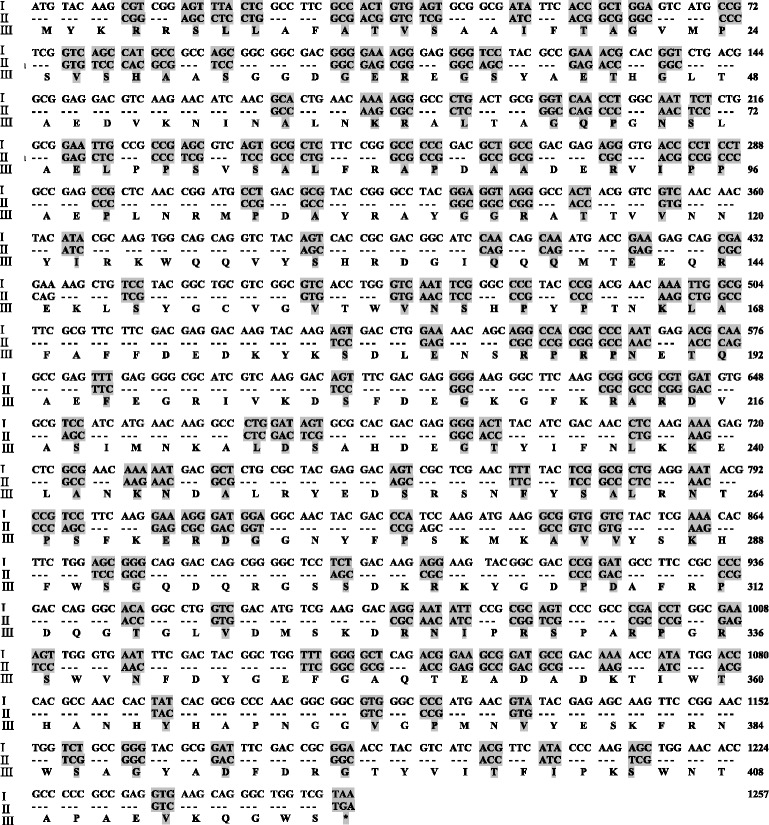
Codon optimization of *S. hygroscopicus* WSH03-13 TGase. The Greek numerals (left side) indicated the sequence type of TGase: I: original sequence of *S. hygroscopicus* TGase ORF; II: *Streptomyces* preferred gene sequence of the TGase ORF; III: amino acid sequence of the TGase ORF. The grey shadows and “---” indicated the mutated positions and invariant positions. The numbers (right side) indicated the nucleotide sequences

**Fig. 4:**
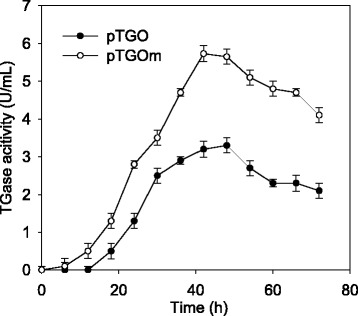
The effect of codon optimization on the expression of TGase in *S. lividans*. The plasmid pTGO encoded TGase ORF with the optimized promoter (see Fig. 2b). The plasmid pTGOm encoded the same promoter as pTGO, and the TGase ORF was optimized according to the codon preference of *S. lividans* (see Fig. 3). The recombinant *S. lividans* TK24 that expressed pTGO or pTGOm was inoculated into 30 mL of medium (which contained 50 μg/mL apramycine) and cultured at 30 °C and 200 rpm for 3 days

To improve the TGase expression in *S. lividans*, the gene sequence of TGase ORF was optimized according to the gene codon bias of *Streptomyces* and was chemically synthesized (Fig. 3). The codon-optimized TGase ORF along with the intact upstream (−48 to −1) and downstream (1258 to 1715) was then cloned into the *Sph* I-*Bgl* II sites of pTGO, which yielded pTGOm. As shown in Fig. 4, when *S. lividans* expressed pTGOm, the highest yield of TGase (5.73 U/mL) was obtained, which was 73.6 % higher than that produced by *S. lividans* when it harbored pTGO. Moreover, the former recombinant strain achieved the highest yield of TGase at 42 h, while for the latter strain, the highest yield was obtained at 48 h. Consequently, the productivity of *S. lividans* when it expressed pTGOm was 0.14 U/mL/h, which was twofold higher than that of *S. lividans* while it harbored pTGO.

## Discussion

Although TGase from *Sv. ladakanum* [14] and *S. platensis* [15] has been expressed in *S. lividans* JT46 by TGase promoters, the yields of TGase reach only 1.23–2.22 U/mL after 3–6 days of fermentation, and the productivities are less than 0.03 U/mL/h [14, 15]. Recently, another recombinant *S. lividans* has obtained only 0.07 U/mL/h of TGase productivity by using the *Streptomyces cinnamoneus* phospholipase D promoter and signal peptide [21]. In this study, *S. lividans* TK24 that harbored pTGM obviously obtained a higher yield (5.73 U/mL) and productivity (0.14 U/mL/h) for the TGase (Fig. 4). It has been reported that different proteases showed variant activation efficiencies against *Streptomyces* pro-TGase in vitro [22]. Because all of these TGases are expressed in pro-TGase form, *S. lividans* TK24 could have those proteases that are more favorable for the pro-TGase activation in contrast to *S. lividans* JT46 [23].

To improve the production of TGase, the endogenous promoter of *S. hygroscopicus* TGase was engineered. Previously, we isolated a TGase-producing strain *S. hygroscopicus* WSH03-13 and cloned the TGase ORF with a flanking sequence [10, 20]. According to the sequence analysis, the upstream sequence of the ORF contained a putative promoter [10]. However, the efficiency and the exact site of this endogenous promoter were not clear. Expression of the TGase ORF with the upstream sequence obtained extracellular TGase activity in *S. lividans*, confirming the existence of the endogenous promoter (Fig. 1). Based on deletion analysis, the complete sequence of the TGase endogenous promoter is restricted to the region from −693 to −48, and a negative element (−198 to −148) was identified (Fig. 2). Finally, the TGase production in *S. lividans* was increased by 81.3 % through the deletion of this element (Fig. 2b). Further investigation should be focused on the action mode of the negative element, which may serve to understand the physiological function of the TGase in *Streptomyces*.

Codon optimization was also used to improve the TGase expression in *S. lividans*. There is evidence of improved expression in the host strain when certain rare codons are replaced with preferred codons [24–26]. This phenomenon is thought to be related to the relative levels of the intracellular pool of charged transfer RNA molecules, which are low for rare codons and high for abundant codons [27]. As indicated by our previous study [10], *S. hygroscopicus* TGase ORF contains a rare codon TTA (leucine, codon usage 0.2 %) in *Streptomyces* [28]. Thus, it could prevent TGase expression because of the low level of transfer RNA molecules. In this study, the gene sequence optimization of TGase ORF according to the codon bias of *Streptomyces* resulted in 73.6 % enhanced TGase production in *S. lividans*. To be noted, the codon optimization reduced the fermentation period for the highest TGase activity by 6 h (Fig. 4). After the sequence optimization, TTA that encoded Leu in the TGase ORF was mutated to the *Streptomyces* preferred codon CTC (Fig. 3). Because *bldA,* which encodes tRNA(Leu) (UUA), has been reported to be expressed only during the late stage of growth [28], the replacement of TTA by the *Streptomyces* preferred codon could account for the reduced fermentation period of the cells that expressed optimized TGase gene.

To further increase the production level of recombinant TGase at a large scale, the optimization medium and culture conditions will be performed in fermentors [29, 30].

## Conclusions

In conclusion, we constructed an active TGase-producing strain with a high yield and productivity, which could be a good candidate strain for industrial production of this enzyme. Moreover, the optimized TGase promoter and site-directed of rare codon TTA may also useful for improving other protein expression in *S. lividans*.

## Methods

### Bacterial strains, plasmids, and culture conditions

*S. hygroscopicus* WSH03-13 that produces TGase was stored in our lab [10]. *E. coli* JM109 was used for gene cloning. *S. lividans* TK24 (*Str*-6, tipAp induced, SLP2-, SLP3-) and pIJ86 (*Streptomyces* complementation plasmid; oriColE1 SCP2* aac(3)IV ermE*p) were used as the expression host and plasmid, respectively. *Streptomyces* cultures were grown on R2YE agar [31] or in liquid that contained glycerol 20 g/L, peptone 20 g/L, yeast extract 5 g/L, MgSO_4_ 2 g/L, K_2_HPO_4_ 2 g/L, KH_2_PO_4_ 2 g/L, and CaCl_2_ 1 g/L. A loop of fresh spore suspension of *S. hygroscopicus* WSH03-13 or *S. lividans* TK24 was inoculated into 30 mL of medium and cultured at 30 °C and 200 rpm for 2–3 days. *E. coli* JM109 was grown in Luria-Bertani medium at 37 °C.

### Construction of plasmids that express TGase with their endogenous promoter

To obtain a plasmid that expresses TGase with its endogenous promoter, a 2.6-kb DNA fragment (*tg1*) that contained the TGase gene with a flanking sequence from *S. hygroscopicus* WSH03-13 was amplified by PCR using the primer pairs TGUF/TGDR (Table 1), and the fragment was then inserted into the *Kpn* I-*Bgl* II sites of pIJ86, which resulted in the plasmids pIJ86/*tg1* (Fig. 1a).

**Table 1:** Primers used in this study

Primer	Sequence (5′–3′)
TGUF	CGGGGTACCCCGTAGCGGGTGGCGAAGAT
TGDR	GGAAGATCTCACGAGGACACCGAACGACTG
TG100F	TTCGAGCTCGGTACCCACCCCGCTGAATGGGACTCTTCGT
TG200F	TTCGAGCTCGGTACCCCAGGAGCAGGGGAACGCTGC
TG300F	TTCGAGCTCGGTACCGACGTTGCCGGGGAGTTGGCGC
TG400F	TTCGAGCTCGGTACCCTCTCCCTGCGGTCGCCGTGACAG
TGQ1R	ACATGCATGCGACCTCAGCCGCGCTGTCCTGGGTC
TGQ2R	ACATGCATGCCCGCCACGAGGCGGAAGGAGATGC
TGQ3R	ACATGCATGCGCGTGGCCGTCGCCGGTCATGACCTGGTG
TGQ4R	ACATGCATGCGGCGGCACCGGTGCCTCGCTACATC
TGQ5R	ACATGCATGCGGGCCCGGTCCGGGGGCCGAGG
TGQ6R	ACATGCATGCGGGAGTGCATGAAGTCGGTGTC
TGQ7R	ACATGCATGCACAGCGGCGGTCGCCGGGGCGACGG
TGQ8R	ACATGCATGCGCCTCGCCGCGAACCGCACGCCAGG
TGQ9R	ACATGCATGCGGCAGGTCGGGAGCGCCTGTC

### Construction of plasmids that express TGase with partially deleted endogenous promoters

To partially delete the 5′-end of the promoter region, each gene fragment of *tg1* with a 5′-end deletion at the promoter region (Fig. 2a) was amplified from pIJ86/*tg1* by PCR using a specific forward primer and a constant reverse primer (TGDR) (Table 1). For the deletion of the first 100 bp nucleotides at the 5′-end of the promoter region, TG100F (Table 1) was used as a forward primer. For further deletions at the 5′-end of the promoter region, TG200F, TG300F, and TG400F were in turn used as the forward primer (Table 1). The resulting PCR products were inserted into the *Kpn* I-*Bgl* II sites of pIJ86 to produce pTGU2, pTGU3, pTGU4, and pTGU5, respectively (Fig. 2a).

To partially delete the 3′-end of the promoter region, the gene fragment that contained the complete open reading frame (ORF) of TGase and the fragments that encoded the promoter region with 3′-end deletions were amplified from pIJ86/*tg1* by PCR, separately (Fig. 2b). Each gene fragment that encoded the promoter region with the 3′-end deletion was obtained by using a constant forward primer (TGUF) and a specific reverse primer (Table 1). For the deletion of the first 50 bp nucleotides at the 3′-end of the promoter region (Fig. 2b), TGQ1R (Table 1) was used as a reverse primer. For further 3′-end deletion of the promoter, TGQ2R, TGQ3R, TGQ4R, TGQ5R, TGQ6R, TGQ7R, TGQ8R, and TGQ9R were in turn used as the forward primer (Table 1). The resulting PCR products were inserted into the *Kpn* I-*Sph* I sites of pIJ86/*tg1* to produce pTGD2, pTGD3, pTGD4, pTGD5, pTGD6, pTGD7, pTGD8, pTGD9, and pTGD10, respectively (Fig. 2b).

The negative element (−197 to −149) was removed from the TGase promoter (−693 and −48) by chemical synthesis, and the resulting gene fragment was cloned into the *Kpn* I-*Sph* I sites of pIJ86/*tg1* to produce pTGO (Fig. 2b).

### Codon optimization of the TGase gene in *S. lividans*

According to the codon preference of *S. lividans*, the *S. hygroscopicus* TGase ORF was optimized and synthesized by Genscript (Nanjing, China). The codon-optimized TGase ORF with an intact upstream (−48 to −1) and downstream (1258 to 1715) was then cloned into the *Sph* I-*Bgl* II sites of pTGO, which yielded pTGOm.

### Expression of the TGase gene in *S. lividans*

Molecular methods for *Stretomyces* were used as described by Hopwood et al. [31]. Plasmids that expressed TGase with an endogenous promoter or its partially deleted versions were transformed into *S. lividans* TK24. The *S. lividans* transformants were selected on a plate that contained 50 μg/mL apramycine. When the transformants were grown in liquid medium, 50 μg/mL apramycine were added. The recombinant *S. lividans* TK24 were inoculated into 30 mL of medium and cultured at 30 °C and 200 rpm for 2–3 days.

### Assay of TGase activity

TGase activity was measured using a colorimetric procedure in which N-α-carbobenzoxyl-glutaminyl-glycine (N-CBZ-Gln-Gly) (Sigma, Shanghai, China) was used as the substrate [8]. Forty microliters of substrate solution (30 mmol/L N-CBZ-Gln-Gly, 100 mmol/L hydroxylamine, 10 mmol/L glutathione, 200 mmol/L Tris-HCl buffer, pH6) was added to 100 μL of TGase solution to initiate the enzymatic reaction. After 10 min, the reaction was stopped by the addition of a 40-μL terminator (1 mol/L HCl, 4 % (v/v) trichloroacetic acid, 2 % (m/v) FeCl_3_ · 6H_2_O), and the reaction solution was subjected to spectrophotometry analysis at 525 nm. A calibration curve was obtained using L-glutamic acid γ-monohydroxamate (Sigma, Shanghai, China). One unit of TGase was defined as that required to generate 1 μmol of L-glutamic acid γ-monohydroxamate per min at 37 °C.

### Protein analysis

SDS-PAGE was performed on a 12 % running gel, and the resolved proteins were visualized by staining with Coomassie Brilliant Blue R250. Protein concentrations were measured using the Bradford method, with bovine serum albumin as the standard.

## Abbreviations

N-CBZ-Gln-Gly: N-α-carbobenzoxyl-glutaminyl-glycine
ORF: complete open reading frame
TGase: transglutaminase
